# Ten years after introduction of NADC30-like strain in China: a novel chimeric porcine reproductive and respiratory syndrome vaccine candidate

**DOI:** 10.3389/fimmu.2025.1585197

**Published:** 2025-05-08

**Authors:** Weixin Wu, Xinyu Fang, Yiyao Jiang, Jiameng Hu, Qiongqiong Zhou, Peng Gao, Yongning Zhang, Xinna Ge, Jun Han, Xin Guo, Lei Zhou, Hanchun Yang

**Affiliations:** ^1^ National Key Laboratory of Veterinary Public Health Safety, College of Veterinary Medicine, China Agricultural University, Beijing, China; ^2^ Key Laboratory of Animal Epidemiology of Ministry of Agriculture and Rural Affairs, College of Veterinary Medicine, China Agricultural University, Beijing, China

**Keywords:** porcine reproductive and respiratory syndrome virus, chimeric, reverse genetics, vaccine, NADC30-like strain

## Abstract

**Introduction:**

Porcine reproductive and respiratory syndrome (PRRS), caused by the PRRS virus (PRRSV), is an economically significant swine disease with extensive strain variation and limited heterologous protection. Modified live virus (MLV) vaccines developed by serially passaging the virus in monkey kidney cell lines have been widely used for more than 20 years. Lineage 1 virus, such as NADC30-like in China and L1C 1-4-4 strains in the United States, have gradually become the predominant strain or the dominant recombination isolate donor strain in recent years. MLVs licensed for use in the market supply low efficacy of heterologous protection ability against the NADC30-like strain, and a vaccine with improved safety and efficacy is therefore required. The method of virulence attenuation used for classical strains may not be applicable to the development of a vaccine against NADC30-like strains due to their low fidelity of replication.

**Methods:**

Chimeric RvBJ-4-(ORF2-4)SX, RvBJ-4-(ORF5-6)SX, and RvBJ-4-(ORF2-6)SX were constructed by substituting minor structural proteins (GP2, GP3, and GP4), major structural proteins (GP5 and M) or both in NADC30-like CHsx1401 to classical strain backbone BJ-4. RvBJ-4-(ORF2-6)SX. Animal trials were conducted to assess the pathogenicity and protection of chimeric viruses.

**Results and Discussion:**

Chimeric virus RvBJ-4-(ORF2-6)SX demonstrates a favorable balance between safety and efficacy, with limited pathogenicity and providing faster viremia clearance as well as reduced lung lesions in vaccinated/challenged pigs. A novel strategy for providing safe and effective immunological protection against NADC30-like strains has been introduced, with the potential for implementation in the field.

## Introduction

Porcine reproductive and respiratory syndrome (PRRS) is a significant disease that poses a considerable threat to the health of pig herds, inducing reproductive disorders in breeding pigs and respiratory diseases in pigs of all ages, leading to serious secondary bacterial or viral infections. The mortality rate of infection with highly pathogenic strains can reach up to 100% in pigs (1, 2). The causative pathogen PRRS virus (PRRSV) was classified as *Betaarterivirus europensis* (known as PRRSV-1) and *Betaarterivirus americense* (known as PRRSV-2) in *Betaarterivirus* Genus according to the International Committee on Taxonomy of Viruses (ICTV) release v4, October 30, 2024 (https://ictv.global/taxonomy/).

The genome of PRRSV is approximately 15 kb and contains at least 10 identified open reading frames (ORFs). ORF1a and ORF1b encode viral replicase polyproteins pp1a and pp1ab, which are further proteolytically processed into at least 12 nonstructural proteins (nsps). ORFs 2–5 encode the glycoprotein GP2-GP5, ORF6 encodes the membrane proteins (M), ORF7 encodes the nucleocapsid protein (N), ORF2a encodes the GP2a protein, ORF2b is located in ORF2a and encodes the small E protein (3), and ORF5a encodes the GP5a protein (4). The viral GP5/M protein forms a dimer that binds to Heparan sulphate (HS) on the surface of porcine alveolar macrophages (PAMs), thereby enriching viral particles to the cell surface and promoting GP5 binding to Sialoadhesin (Sn) to mediate viral internalization (5, 6). Furthermore, GP2a/GP3/GP4 proteins form a trimer through which virus particles entering the endosome interact with CD163 to induce membrane fusion (7). Previous studies have demonstrated that the structural protein (SP) region plays a key role in providing homologous immunity (8–15), in which N protein is conserved within species and has a kissing interaction with the 3’ untranslated region (UTR) (16).

PRRSV has represented a considerable threat to the global pork industry for over three decades (17). The virus was initially identified in mainland China in 1995 (18), and the NADC30-like strain was introduced and circling in Mainland China more than ten years (19, 20). Ongoing clinical surveillance has demonstrated that NADC30-like strains, belonging to lineage 1 of PRRSV-2, have become the primary dominant PRRSV in China, representing a significant threat to the current pig industry (19, 21, 22). Similarly, the lineage 1 virus is also predominant in the United States. At present, modified-live virus (MLV) vaccine remains an important tool for disease prevention and control, even the farms using herd closure strategy for PRRS elimination, prefer to use a safe and effective vaccine to achieve herd immunity before closing herd. However, current commercial MLV vaccines have very limited cross-protection against NADC30-like strains (22–26). In addition to efficacy, safety is another important consideration. MLV derived from the prototype strain VR-2332 was introduced in the United States in 1994 and has been widely used since then with proven clinical safety in experimental and field applications (27).

However, MLV derived from highly pathogenic PRRSV (HP-PRRSV) parental strains shows a high potential for reversion to virulence (28, 29), and the high virulent reversion strains isolated from fields have highlighted their fatal flaws as an acceptable vaccine (30, 31). In addition, an increasing number of recombinant strains between HP-PRRSV MLV reversion strains and NADC30-like strains have been isolated from farms (22, 32–36), but the recombination between VR-2332 derived MLV and NADC30-like strains is limitedly reported. Our recent study indicated that the NADC30-like strains show genomic characters with higher self-recombination and mutation rates, attributed to the low fidelity of the RNA-dependent RNA polymerase (RdRP) (37). It means the traditional approach of attenuating virulence by passaging in heterologous cells may not be applicable to the development of a vaccine against NADC30-like strains.

Several chimeric strains from PRRSV-1, HP-PRRSV, or other strains have been previously developed as vaccine candidates (15, 38, 39), while only one chimeric virus with HP-PRRSV and NADC30-like strains as backbones has been reported until last year (40). However, it is crucial to acknowledge the potential risks of reversion to virulence, as previously highlighted, when employing the HP-PRRSV backbone in chimeric vaccines. In this study, we introduced a chimeric strategy using the lineage 5 strain BJ-4 as the backbone, which is a low virulence strain like VR-2332 and was the first PRRSV isolate sequenced in mainland China in 1997 (41). And substitute the encoding regions of minor envelope proteins (GP2, GP3, and GP4), major envelope proteins (GP5 and M), or together them from a NADC-30 like strain CHsx1401, which was the first NADC30-like strain isolated in China (19). Our research indicated that the BJ-4 can serve as a safe backbone, and the envelope proteins from CHsx1401 can provide great immune-protection compared with the backbone parental strain. And the chimeric strain can serve as a vaccine candidate for follow-up studies.

## Results

### Chimeric viruses were successfully rescued with different viral proliferation abilities *in vitro*

**Figure 1:**
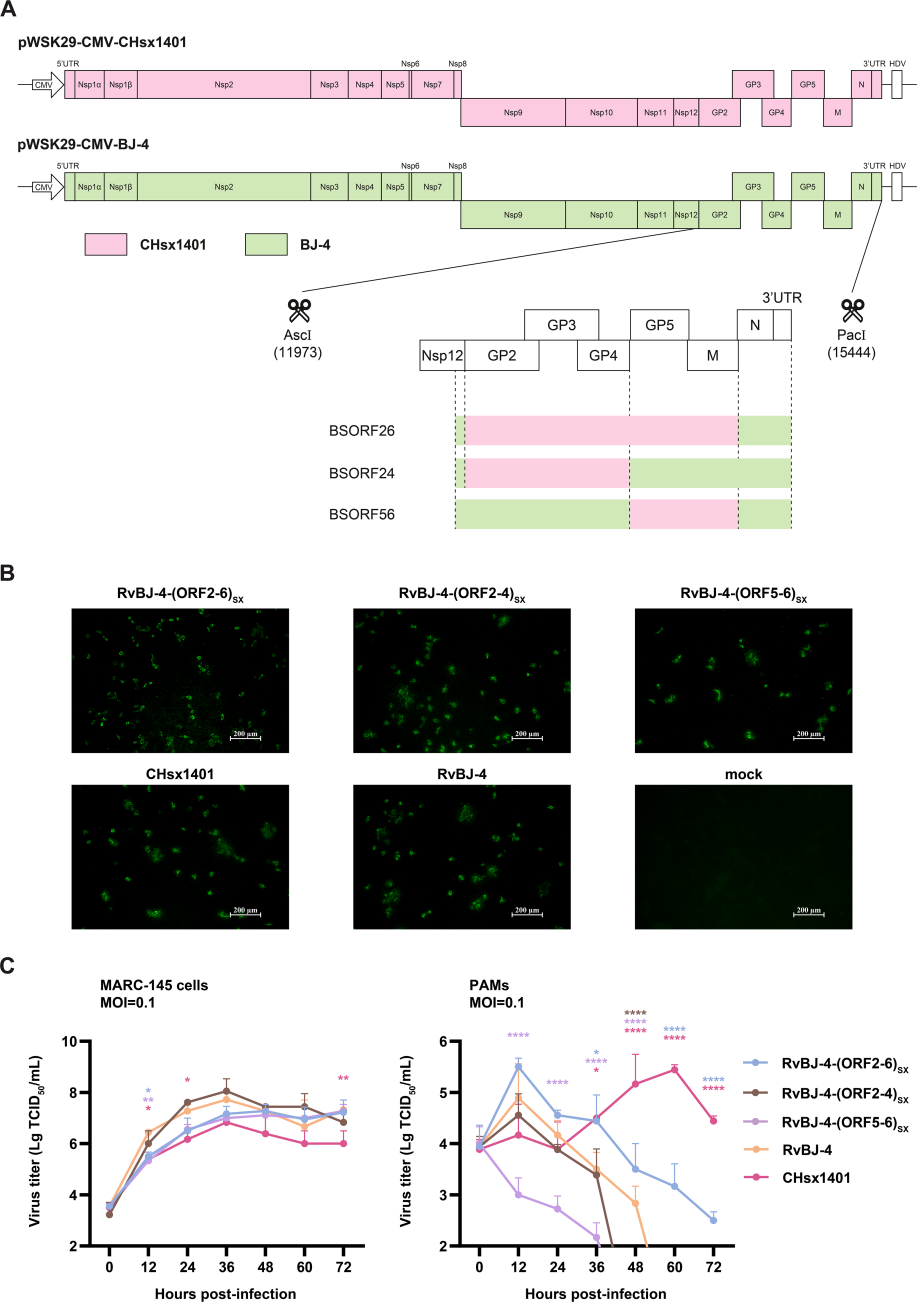
Constructing of full-length cDNA clones by segment and fusion PCR **(A)**, followed by enzyme digestion and homologous recombination. Different segments for fusion PCR are separated by dotted lines. Rescued virus and CHsx1401 were identified using IFA in MARC-145 cells **(B)**. Multistep growth kinetics of rescued viruses were performed in MARC-145 cells or PAMs **(C)**. Asterisks indicate statistical significance between RvBJ-4 and other strains are shown in different colors; NS, no significance; *P* < 0.05; **, *P* < 0.01; ****, *P* < 0.0001.

The plasmids of the full-length infectious clone were constructed by using fusion PCR and homologous recombination (**Figure 1A**). Chimeric viruses and their parental backbone virus were successfully rescued and designated as RvBJ-4-(ORF2-4)_SX_, RvBJ-4-(ORF5-6)_SX,_ BJ-4-(ORF2-6)_SX_ and RvBJ-4 (**Figure 1B**). The growth kinetics of the chimeric virus and parental viruses were evaluated by infecting MARC-145 cells or PAMs at 0.1 MOI (**Figure 1C**). For the parental strains, RvBJ-4 proliferates faster in MARC-145 cells and has significantly higher peak titer (7.72 ± 0.25 Lg TCID_50_/mL) than CHsx1401 (6.83 ± 0.29 Lg TCID_50_/mL) at 36 hour post inoculation (hpi), but had lower peak titer in PAMs (4.89 ± 0.67 Lg TCID_50_/mL at 12 hpi versus 5.44 ± 0.10 Lg TCID_50_/mL at 60 hpi). With respect to the chimeric virus, RvBJ-4-(ORF2-4)_SX_ has an even higher peak titer (8.05 ± 0.48 Lg TCID_50_/mL) than RvBJ-4 in MARC-145 at 36 hpi, while RvBJ-4-(ORF2-6)_SX_ and RvBJ-4-(ORF2-4)_SX_ are intermediate, indicate a good proliferation ability in vaccine producer cells. RvBJ-4-(ORF2-4)_SX_ has similar growth kinetics to its parental backbone strain RvBJ-4 in PAMs, but with the addition of minor structural proteins, RvBJ-4-(ORF2-6)_SX_ has a peak titer about four times than that of RvBJ-4 (5.50 ± 0.17 Lg TCID_50_/mL versus 4.89 ± 0.67 Lg TCID_50_/mL) in PAMs at 12 hpi, indicate a better proliferation ability in host cells. Intriguingly, RvBJ-4-(ORF5-6)_SX_ with major envelope protein substitution cannot proliferate normally in host cells, but can still grow well in MARC-145 cells. We were curious whether such a “single round infection” virus could lead to good immunological protection. Besides, earlier peak titers of RvBJ-4 and chimeric viruses might indicate an earlier clearance of viremia.

### Pathogenicity and replication ability of chimeric virus shows similarity to BJ-4 backbone *in vivo*

**Figure 2:**
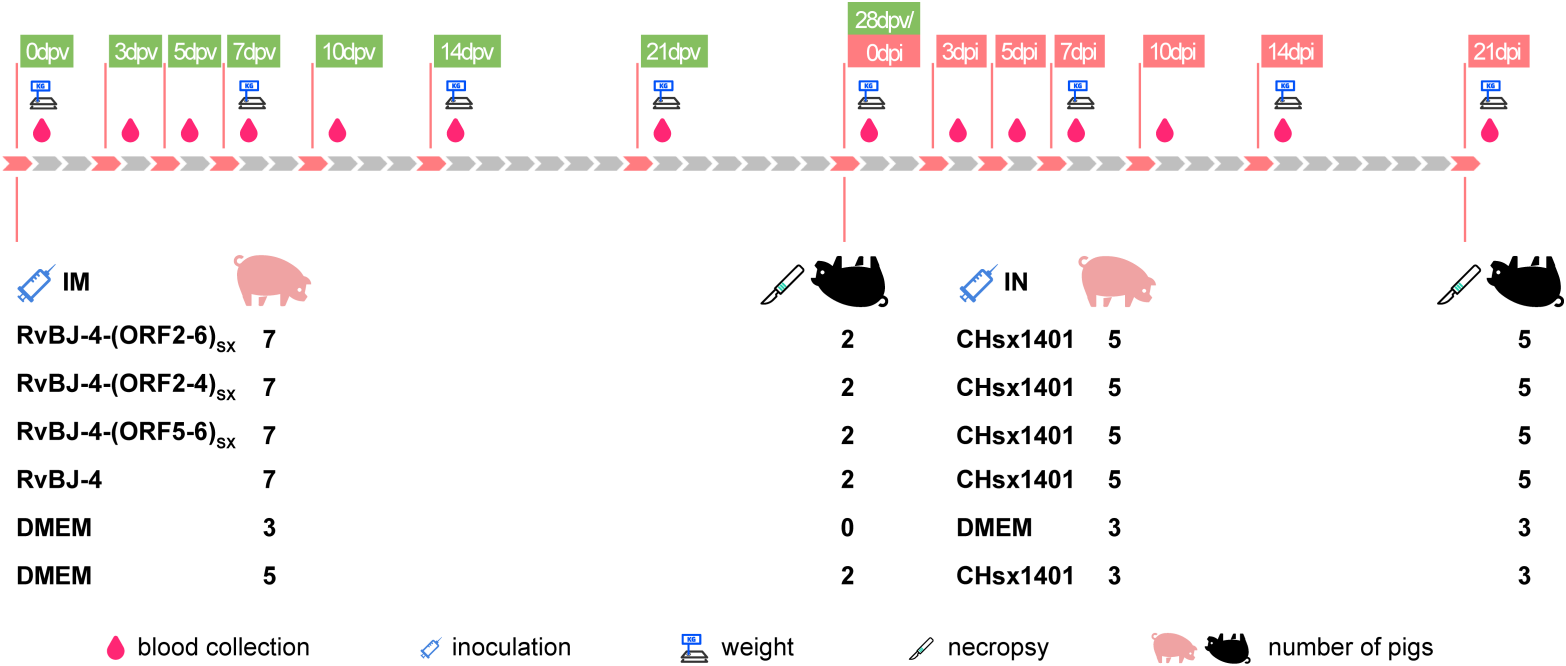
The scheme of the animal trial.

**Figure 3:**
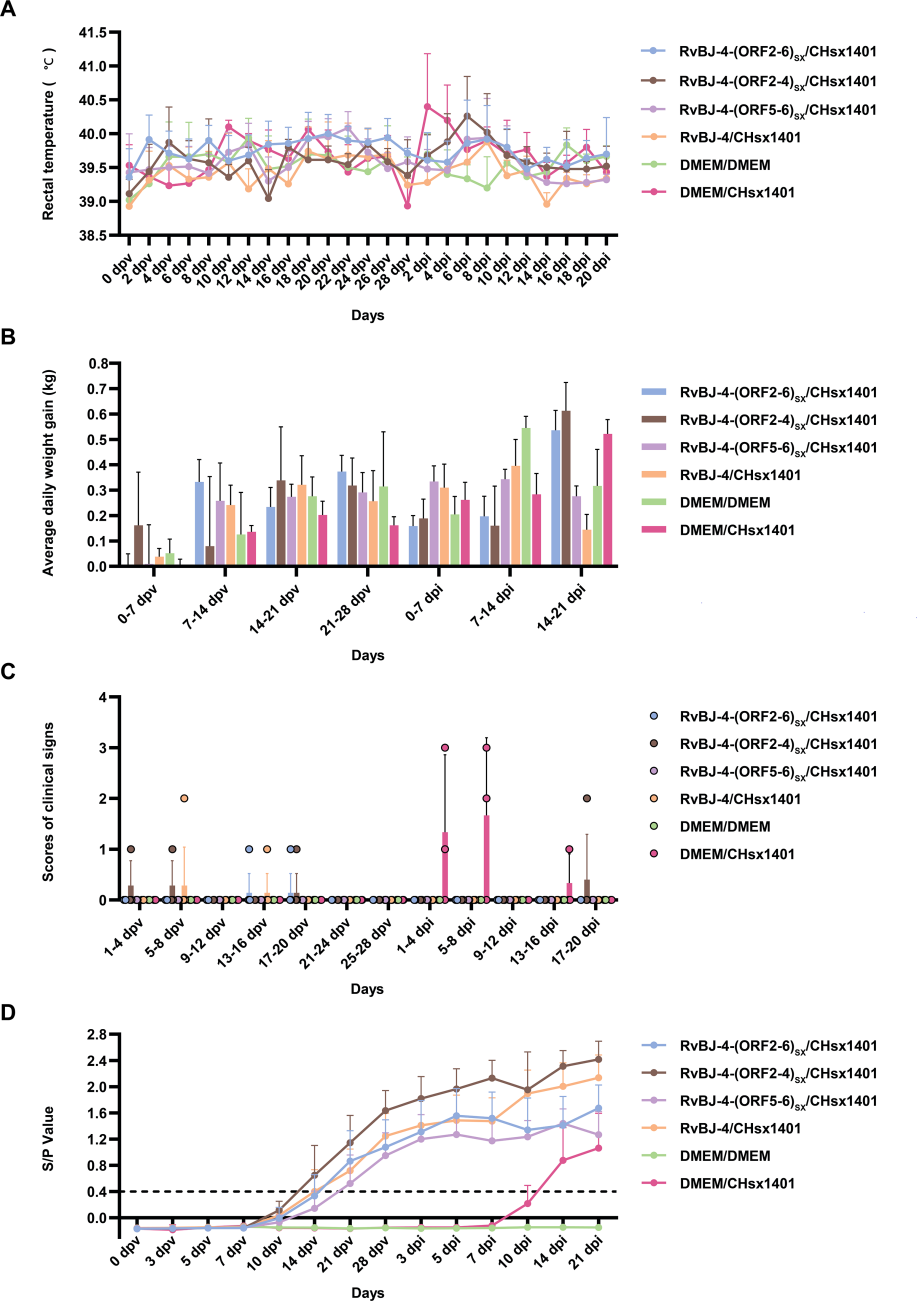
The clinical data of inoculated pigs. The rectal temperatures **(A)**, average daily weight gain (ADG) **(B)**, clinical signs scores **(C)**, and PRRSV-specific antibody kinetics **(D)** of each group are shown as means ± SD (error bars). A ratio of ≥0.4 was regarded as seroconversion.

To examine the influence of substituting CHsx1401 structural protein into the BJ-4 backbone on viral pathogenicity, an animal inoculation experiment was carried out. The 29-day-old specific pathogens-free (SPF) Landrace pigs (**Figure 2**), were intramuscularly vaccinated with 2 mL 10^5^ TCID_50_/mL chimeric virus, parental backbone virus RvBJ-4 or 2 mL cultured MARC-145 cells in DMEM as the negative control, respectively. The pathogenicity of chimeric viruses and their parental virus RvBJ-4 were assessed and recorded as days post-vaccination (dpv) (**Figure 2**). Rectal temperature remained below 40.5°C in each group (**Figure 3A**) and vaccination did not affect the average daily weight gain (ADG) (**Figure 3B**). Two pigs in the RvBJ-4-(ORF2-4)_SX_ group sneezed during feeding at 4 and 6 dpv, as did two pigs vaccinated with RvBJ-4 at 8dpv (**Figure 3C**). Several sneezes were observed during 13–20 dpv in different vaccinated groups and no other clinical signs were observed before the challenge.

**Figure 4:**
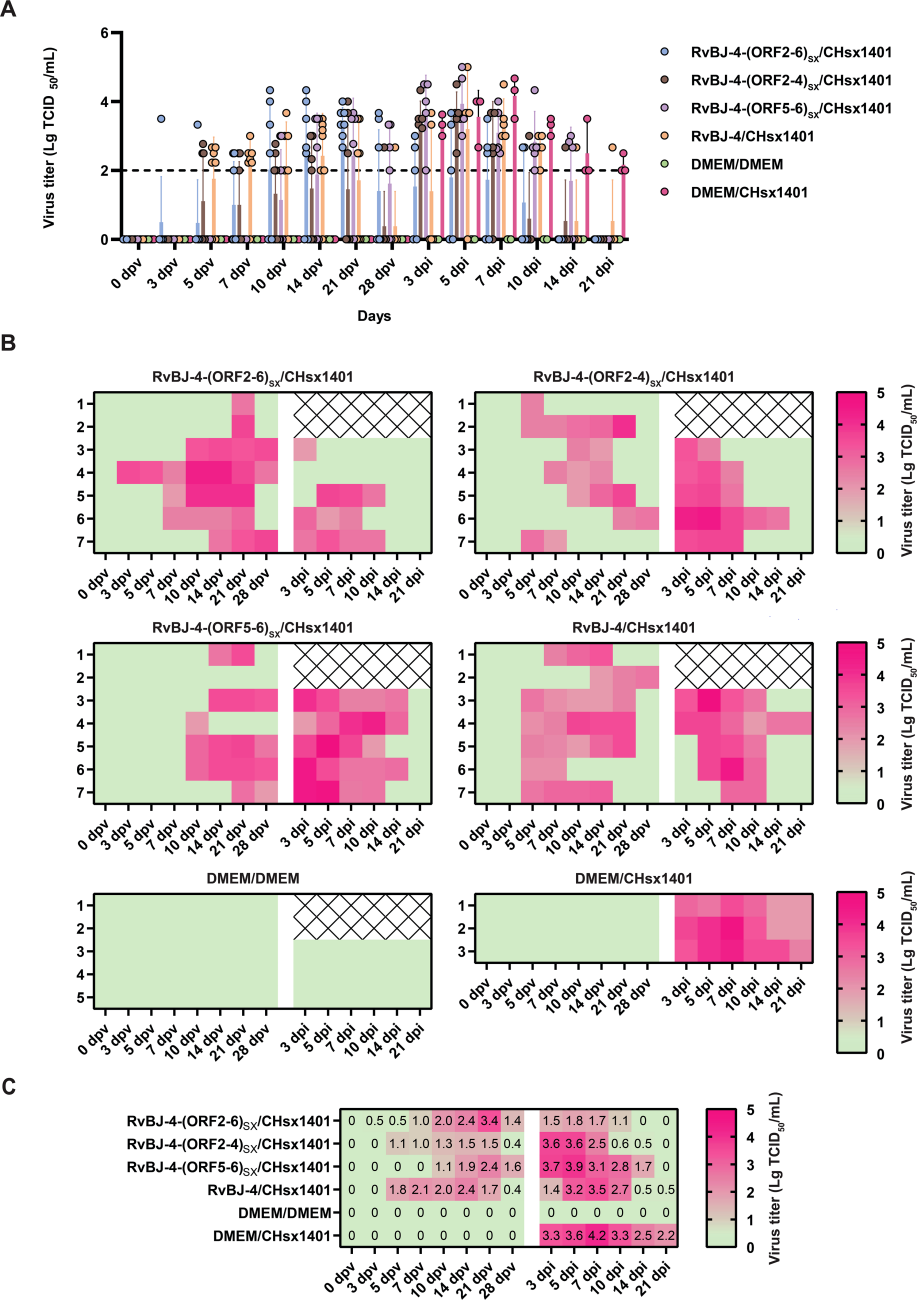
Viremia kinetics in pigs. Data are shown as a histogram with each spot representing an individual **(A)**, a heat map with each line representing an individual at different times **(B)**, and means in different groups **(C)**. The detection limit of virus titer is 10^2^ TCID_50_/mL and the data below are shown as 0.

**Figure 5:**
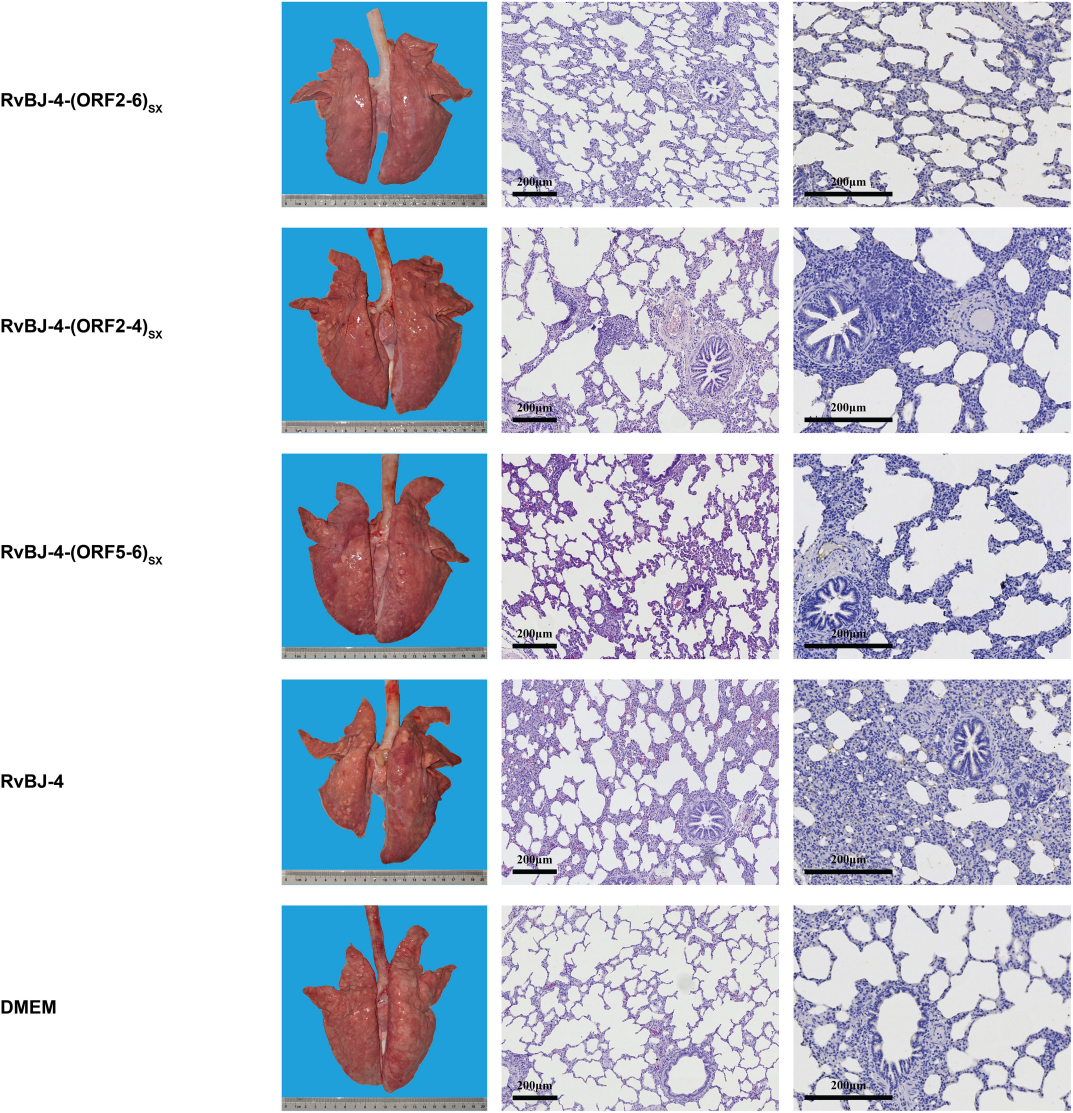
Lung lesions and immunohistochemistry examination in the immune phase. Representative images of gross and microscopic lung lesions are presented.

**Figure 6:**
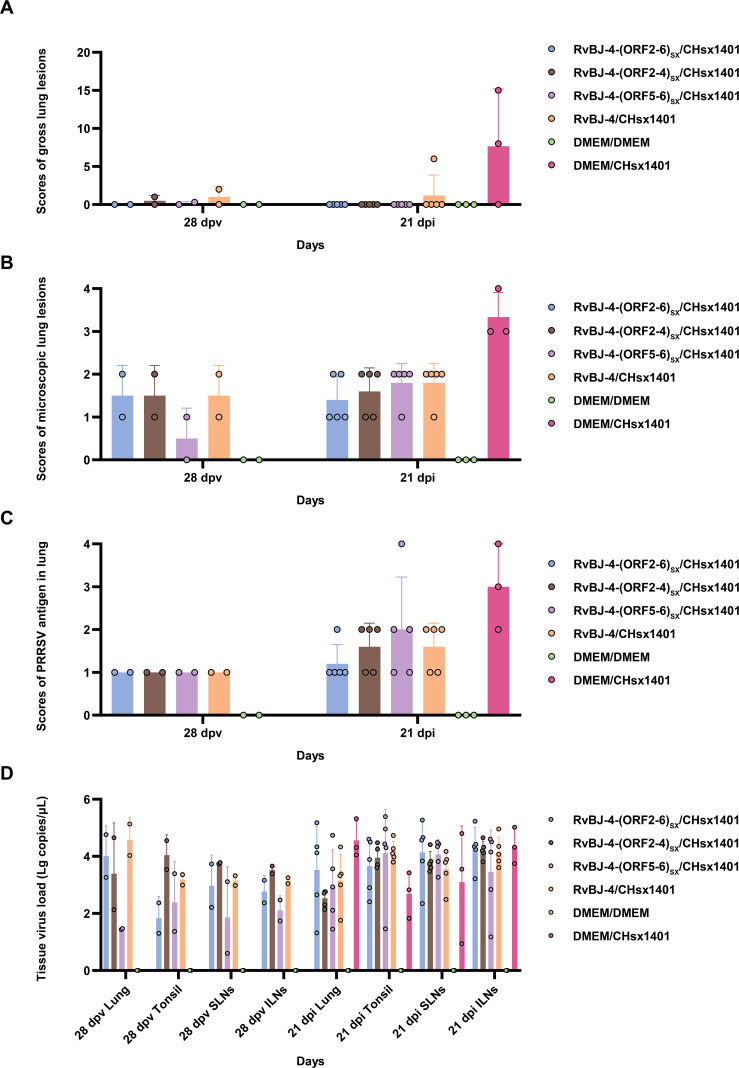
Scores of gross lung lesions **(A)**, microscopic lung lesions **(B)**, PRRSV antigen in the lung **(C)**, and tissue virus load **(D)** were shown as means ± SD (error bars).

PRRSV-specific antibodies against the N protein were converted to positive in most vaccinated groups at 14 dpv, except for RvBJ-4-(ORF5-6)_SX_ showing lower antibody levels than other groups and its seroconversion occurred up to 21 dpv (**Figure 3D**). The virus titer of viremia indicates the growth kinetics of the virus *in vivo*, which shows a large difference among different individuals (**Figures 4A–C**). Most pigs (5/7) in group RvBJ-4 backbone become viremic at 5 dpv, and eliminated (6/7) at 28 dpv, which shows a transient carrying time means less chance to recombine with wild strains (**Figure 4B**). Of all, only one pig in the RvBJ-4-(ORF2-6)_SX_ group became positive at 3dpv and maintained infection at a relatively high level, and two pigs in this group remained negative until 21 dpv. The chimeric virus with minor envelop proteins GP2–4 shows lower viremia levels during vaccination (**Figure 4C**), while pigs in RvBJ-4-(ORF5-6)_SX_ groups did not become positive until 10 dpv, and one of the pigs even remained negative until euthanasia, which was familiar from proliferation kinetics on PAMs *in vitro*, but the ability to proliferate in pigs surprised us. Two pigs from each group, except the positive control (DMEM/CHsx1401), were randomly selected and euthanized for necropsy. Only one pig in the RvBJ-4 group showed a small area of pulmonary consolidation, and there was no obvious severe gross or microscopic lung lesion observed (**Figure 5**). Other pigs showed mild microscopic lesions such as interlobular septal thickening and inflammation (**Figure 5**) and received low lung lesion and PRRSV antigen scores (**Figures 6A–C**). Lungs, tonsils, inguinal lymph nodes (ILNs) and submandibular lymph nodes (SLNs) were collected and processed individually for quantitative analysis of viral tissue load by absolute real-time reverse transcription PCR (RT-qPCR), which showed similar pattern between RvBJ-4 and RvBJ-4-(ORF2-4)_SX_ or RvBJ-4-(ORF2-6)_SX,_ while low level of tissue loads in lung result from RvBJ-4-(ORF5-6)_SX_ (**Figure 6D**).

### Immunization helps alleviate clinical symptoms

After 28 days of vaccination, each pig was intranasally inoculated with 2 mL 10^5^ TCID_50_/mL CHsx1401 virus or 2 mL DMEM medium as a negative control. Cross-protection of the chimeric viruses and their parental virus RvBJ-4 were assessed and recorded as days post-inoculation (dpi). The body temperature of one pig reached 41.3°C at 2 dpi and another reached 40.8 at 4 dpi in the positive control group (**Figure 3A**). There was no obvious pattern among different groups according to ADG (**Figure 3B**). Two out of three pigs in the positive control group sneezed or coughed and all three pigs were inappetent during the first 8 days post-inoculation, but recovered thereafter (**Figure 3C**). One pig in the RvBJ-4-(ORF2-4)_SX_ group started sneezing at 18 dpi, and coughed until 20 dpi. No pigs in the other vaccination groups showed obvious clinical signs such as fever or respiratory signs during challenge. After being challenged with CHsx1401, serum antibodies against N protein gradually increased in the vaccinated groups, and one of the pigs in the positive group became positive at 10 dpi (**Figure 3D**).

### Chimeric viruses conduce to viremia clearance of NADC30-like strain infection

The viremia level of each group is shown in **Figure 4**. Together with the antibody trends, the viremia level further indicated that CHsx1401 had a higher proliferative capacity in pigs compared to chimeric viruses or their backbone strain RvBJ-4. Viremia in the positive control group averaged 10^3.3^ TCID_50_/mL at 3 dpi and peaked at 10^4.2^ TCID_50_/mL on day 7. A similar viremia trend was seen in the RvBJ-4-(ORF5-6)_SX_ group, which means that the substitution of major structural proteins with short-term immune induction cannot play a good protective effect. On the other hand, vaccination with backbone strain RvBJ-4 or chimeric strain RvBJ-4-(ORF2-4)_SX_ partially reduced the viremia maintenance time after challenge, but the average peak virus titer still reached 10^3.5^ TCID_50_/mL at 7 dpi and 10^3.6^ TCID_50_/mL at 3 and 5 dpi, respectively. Surprisingly, the average peak titer of viremia in the RvBJ-4-(ORF2-6)_SX_ group is only 10^1.8^ TCID_50_/mL, which is approximately 100 times lower than in the other vaccination groups. Viremia clearance (virus titer in serum below the detection limit of 10^2^ TCID_50_/mL) in this group was observed at 14 dpi, which means that virus had been cleared from the serum less than 14 days. Moreover, one pig in this group even sustained negative viremia after being challenged, and another pig turned negative just after 5 days. The above results showed that the ability of the NADC30-like strain to replicate in pigs was significantly reduced in vaccinated pigs, especially after vaccination with RvBJ-4-(ORF2-6)_SX_. No virus was detected in the pigs of the negative control group.

### Vaccination alleviates lung lesions caused by NADC30-like strain

**Figure 7:**
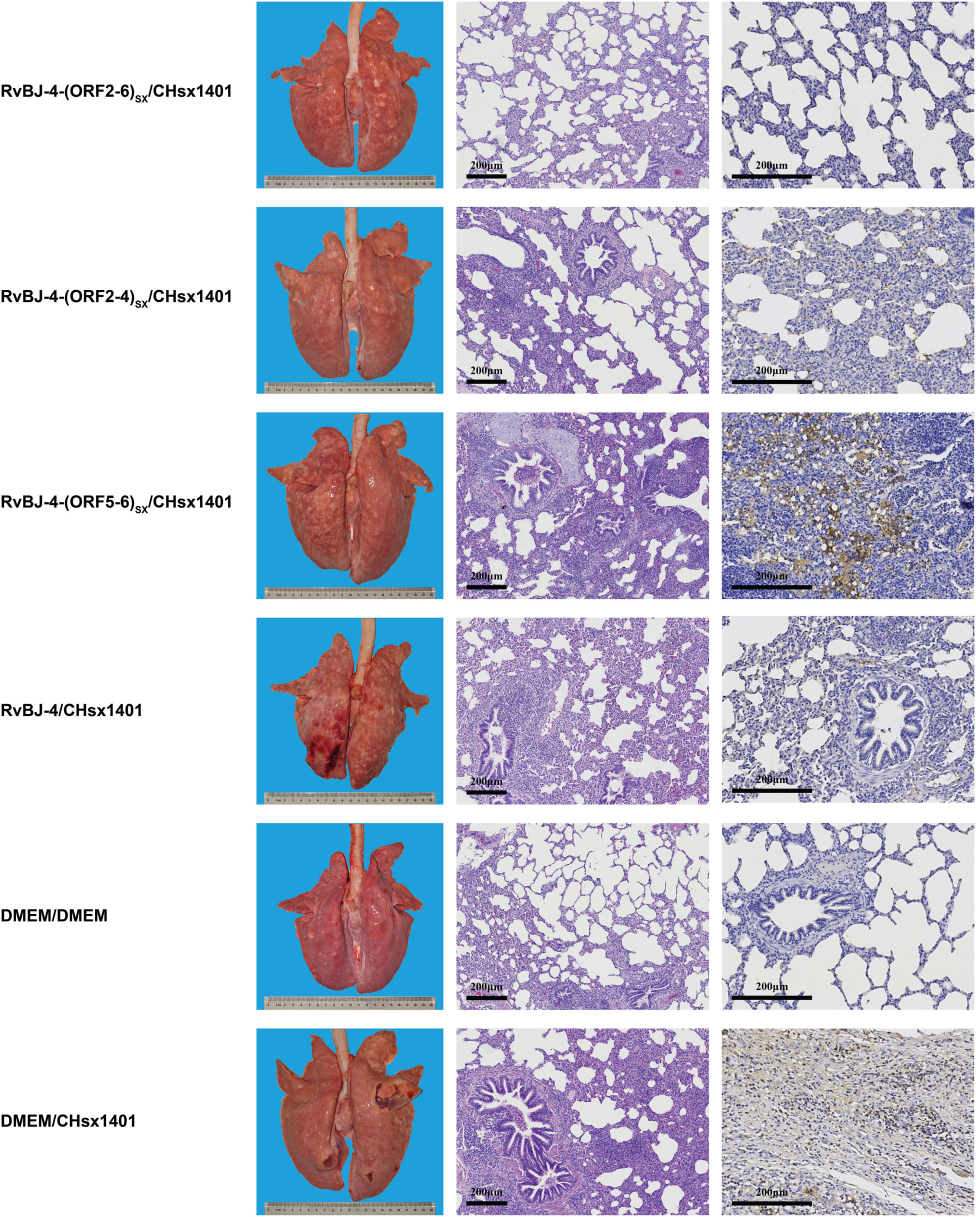
Lung lesions and immunohistochemistry examination in the challenge phase. Representative images of gross and microscopic lung lesions are presented.

All pigs were euthanized and necropsied at 21 dpi, and gross pathology, microscopic pathology, and PRRSV antigen distribution in lungs were assessed subsequently. Without vaccination, CHsx1401-infected pigs presented with typical interstitial pneumonia with pulmonary consolidation, oedema, hemorrhage, and even pulmonary hyperinflation in one pig (**Figure 7**). One of the pigs in the RvBJ-4 group had hemorrhage spots on the surface of the lung, while the rest showed no significant gross pathological injury.

Microscopic lung lesions were further observed after hematoxylin and eosin (H&E) staining, and the distribution of PRRSV antigen in the lungs was examined by immunohistochemistry (IHC) analysis using PRRSV N-specific monoclonal antibody (mAb) (**Figure 7**). The lungs of pigs in the positive control group showed histopathological changes characterized by the extensive disappearance of lung structure, hemorrhage, thickening of the interlobular septa, and infiltration of inflammatory cells and necrotic debris in both the alveolar spaces and bronchioles, as well as mass necrosis in the hyperinflated lung. Immunohistochemical staining showed PRRSV-positive signals filling in the alveolar necrotic structure and septal macrophages around the bronchia, bronchioles, and alveolar septa.

The mean histopathological and immunohistochemical scores were above 3-point in the positive control group, with different numbers of 1-point and 2-point individuals among different vaccination groups (**Figures 6B, C**). The 2-point lungs showed moderate microscopic lesions with a partial disappearance of lung structure, minor hemorrhage, and inflammatory cells with necrotic debris in both alveolar spaces and bronchioles. In addition, there was no obvious pattern in tissue loads (**Figure 6D**). These results suggest that vaccination alleviated lung lesions in pigs.

## Discussion

In 1996, CH-1a, the first strain of PRRSV in mainland China, was isolated from the aborted fetus in a pig farm (18). Science then, several representative strains of PRRSV have been isolated and identified, including BJ-4 (41), HP-PRRSV JXA1 (1) and JXwn06 (42). Around 2013, NADC30-like strains were introduced and quickly became the predominant strain in China, while no fully satisfactory vaccine on the market balances safety and efficacy available against NADC30-like strains for ten years. The invention of MLV faces drawbacks of recombination with field strains hence reversion to virulence, especially in NADC30-like strains due to its low fidelity of replicase protein (37).

In another of our previous studies (43), a highly specific miRNA for PAMs, miR-142, was used by inserting its target sequence into the viral genome to restrict replication exclusively in host cells. Furthermore, in our later study, multiple copies of the miR-142 target sequence were inserted into the PRRSV genome between ORF1b and ORF2a (unpublished data). *In vitro* experiments showed that the chimeric viruses were unable to replicate in PAMs, but the target of miR-142 is partially deleted under selective pressure from the host *in vivo*, allowing them to replicate in pigs and induce pathogenic, even lethal. The classical strain BJ-4 belongs to lineage 5, and the non-structural protein coding region of RvBJ-4 has 99.9% nucleotide homology with the Ingelvac PRRS MLV, which is safer and stabler than HP-PRRSV-derived vaccines. Besides, lineage 5 strains were less involved in the recombination of pathogenic lineage 8 strains (44). In this study, a strategy was attempted by combining the non-structural protein region of the classical strain BJ-4 with the structural protein (including protein E and GP5a) of the NADC30-like strain CHsx1401, expecting they confer better immunological protection. The structural proteins possess antigenic epitopes, which are capable of activating the humoral immune response. The replacement of GP2-GP3-GP4 or GP5-M were separately or together conducted on account of the dimer or trimer structure, and their differing functional roles in the context of infection. The balance between infection damage at the immune stage and its efficacy against challenge is a worthwhile project. The loss of proliferation ability of RvBJ-4-(ORF5-6)_SX_ gives us a model to study the efficacy of “single round infection immune “ against challenge. This was intriguing because the same thing does not happen with the chimeric of CHsx1401 as backbone and JXwn06 as GP5-M donor (unpublished data). The clinical trial of RvBJ-4-(ORF5-6)_SX_ was a complement to the miR142 trial, which did not include the challenge phase. The chimeric of GP5-M shows limited immunological protection against challenges with CHsx1401 during short-term infection at low levels. Reducing viremia and duration of exposure is a good advantage for safety in the immune phase, but the loss outweighs the gain if it is at the expense of efficacy. Therefore, our goal is to improve safety while maintaining efficacy. Our previous study (23) found that the average viremia level was close to 10^1^ TCID_50_/mL with classical MLV after 28 days of vaccination, while it was 10^3^ to 10^4^ TCID_50_/mL in the JXA1 or HB-1 group, indicating that infection with lineage 5 strains is transient. In this study, the chimeric of GP2-GP4 or GP2-M did not alternate the proliferative character in pigs, viremia of these two groups were both less than 10^1.5^ TCID_50_/mL as well as classical strain RvBJ-4 at 28 dpv, and only 1/7 or 3/7 of pigs remain positive at 28 dpv. In contrast, vaccination with the donor strain CHsx1401 resulted in no clearing viremia in all pigs and an average viremia higher than 10^2^ TCID_50_/mL at 28 dpv, as previously described (45).

NADC30-like strains can cause widespread abortion and stillbirth in pregnant sows, but mediate or slight respiratory pathogenetic to pigs (19). However, young pigs on the farm can act as a “reservoir”, “expanding container” and “mixer”, to keep strains circulating on the farm, amplifying the population and recombining with other strains. Therefore, viremia clearance time remains an important benchmark. Each vaccination group in this study shows no significant clinical signs or pulmonary pathology after inoculation with CHsx1401. Whereas the structural protein chimera contributes to the clearance of viremia against CHsx1401. The clearance rate of pigs in the RvBJ-4-(ORF2-6)_SX_ and RvBJ-4-(ORF2-4)_SX_ groups reached 3/5 and 4/5 at 10 dpi, and all viremia was cleared at 14 dpi and 21 dpi. One pig in the RvBJ-4-(ORF2-6)_SX_ group, which had the longest and highest viremia period, was not even detectable for positive viremia after challenge, and another pig returned negative only 5 days after inoculation. The above results show that the chimeric protein contributes greatly to the clearance of viremia, and such a chimeric virus can effectively reduce the duration and level of viremia after being challenged, thus further reducing the risk of recombination.

An ideal vaccine strain should be able to have limited replication in the host to reduce virulence and less chance to recombination with wild strains, but broadly induce an immune response to eliminate the wild strain, in addition to robust proliferation in the cell culture system to reduce production costs. Our study has provided a new strategy for PRRSV vaccine development. The safety and efficacy of this chimeric strain have been verified in our study, and it shows a well-balanced state. It also shows good proliferation ability in MARC-145 cells, indicating a good potential for production as a vaccine. The risk of recombination has been reduced by the rapid clearance of viremia, while the property of chimeric strains to recombine with wild strains and pathogenicity in sows deserves further investigation.

## Materials and methods

### Cells, viruses, and plasmid

Primary PAMs were prepared from 4-week-old specific-pathogen-free (SPF) landrace pigs and cultured in RPMI 1640 medium (Gibco, #61870044) with 10% FBS (Gibco, #16140071) as previously described (46). For PRRSV proliferation and titration, MARC-145, an African green monkey kidney epithelial cell line derived from MA104, was cultured in Dulbecco’s modified Eagle’s medium (DMEM) (Gibco, #12491015) with 10% FBS and penicillin (50 U/mL)-streptomycin (50 μg/mL) at 37°C with 5% CO_2_. HEK-293T cells used for transfection of full-length cDNA were cultured in the same manner as MARC-145 cells. The full-length sequence of BJ-4 was produced using the infectious clone sequence described previously (47) (GenBank accession number EU360128) with an additional T11970G mutation to introduce the Asc I enzyme cleavage site. The infectious clones of BJ-4 and CHsx1401 (GenBank accession number KP861625) were constructed from the plasmid pWSK29-CMV-MCS-HDV-SV40 (48), and stored in our laboratory, named pWSK29-CMV-BJ-4 and pWSK29-CMV-CHsx1401.

### Construction of full-length chimeric cDNA clones

The strategy for the construction of chimeric viruses RvBJ-4-(ORF2-6)_SX_, RvBJ-4-(ORF2-4)_SX_, RvBJ-4-(ORF5-6)_SX_ were illustrated in **Figure 1**. In detail, the full-length cDNA clones of BJ-4 were cut by restriction enzyme *Asc* I and *Xho* I. The insertion fragments of three chimeric viruses were all amplified by overlapping PCR with three fragments each separated by dotted lines, respectively. Each fragment with upstream and downstream flanking sequences was amplified using KOD One™ PCR Master Mix (TOYOBO, #KMM-201) and purified using a Gel Extraction Kit (OMEGA, #D2510). Overlapping PCR was used to amplify the purified products to generate fragments BSORF24, BSORF56, and BSORF24 with BJ-4 upstream and downstream flanking sequences. Homologous recombination between the BJ-4 backbone and the inserted chimeric segments was performed using the ClonExpress Ultra One Step Cloning Kit (Vazyme, #C112-02), followed by transformation of the recombinant plasmid, screening, propagation and plasmid extraction (Promega, #A2492). The primers used to generate the chimeric plasmids are listed in **Table 1**.

**Table 1:** Primers and probe used in this study.

Primer^a^	Sequence (5’-3’)^b^	Usage
BSORF24-1F	CAATGATGCGTTTCG**GGCGCGCC**AGGAAGGGAAAA **(*Asc*I)**	Construction of chimeric cDNA clone pWSK29-CMV-BSORF24
BSORF24-1R	AAGCCCCCATTTCATTTCAATTCAGGCCTAA	
BSORF24-2F	TAGGCCTGAATTGAAATGAAATGGGGGCTTT	
BSORF24-2R	GCATTTCTCCAACATATCTAAACATTCAAATTGCCAACAG	
BSORF24-3F	TTTGAATGTTTAGATATGTTGGAGAAATGCTTGACCGCGG	
BSORF24-3R	GGGACCATGCCGGCC**TTAATTAA**TTTTTTTTTTTTTTTTTTTTTTTTT **(*Pac*I)**	
BSORF56-1F	CAATGATGCGTTTCG**GGCGCGCC**AGGAAGGGAAAA **(*Asc*I)**	Construction of chimeric cDNA clone pWSK29-CMV-BSORF56
BSORF56-1R	AACATATCTAAACATTCAAATTGCCAACAGAATGGCAAAA	
BSORF56-2F	CTGTTGGCAATTTGAATGTTTAGATATGTTGGGGAAATGC	
BSORF56-2R	GTTGTTATTTGGCATATTTAACAAGGTTCACCACTCCTCG	
BSORF56-3F	TGAACCTTGTTAAATATGCCAAATAACAACGGCAAGCAGC	
BSORF56-3R	GGGACCATGCCGGCC**TTAATTAA**TTTTTTTTTTTTTTTTTTTTTTTTT **(*Pac*I)**	
BSORF26-1F	CAATGATGCGTTTCG**GGCGCGCC**AGGAAGGGAAAA **(*Asc*I)**	Construction of chimeric cDNA clone pWSK29-CMV-BSORF26
BSORF26-1R	AAGCCCCCATTTCATTTCAATTCAGGCCTAA	
BSORF26-2F	TAGGCCTGAATTGAAATGAAATGGGGGCTTT	
BSORF26-2R	GTTGTTATTTGGCATATTTAACAAGGTTCACCACTCCTCG	
BSORF26-3F	TGAACCTTGTTAAATATGCCAAATAACAACGGCAAGCAGC	
BSORF26-3R	GGGACCATGCCGGCC**TTAATTAA**TTTTTTTTTTTTTTTTTTTTTTTTT **(*Pac*I)**	
PRRSV-2-qPCR-F	GTACATTCTGGCCCCTGCC	Detection of PRRSV-2
PRRSV-2-qPCR-R	TTCTGCCACCCAACACGAG	
PRRSV-2-qPCR-probe	FAM-ATAACCACGCATTTGTCGTCCGGCGT-BHQ1	

a F denotes forward PCR primer; R denotes reverse transcription or reverses PCR primer.

b Restriction enzyme sites introduced by PCR are shown in boldface and specified in parentheses at the end of the sequence.

### Recovery of chimeric virus

HEK-293T cells were transfected with 1.25μg infectious plasmids and pCAGGS-HA-CD163 (prepared and stored in our laboratory) at a density of approximately 70% using the LTX (Invitrogen, #15338100) in 6-well plates. The culture was replaced with 2% DMEM medium 12 hours after transfection. The supernatant of the cell culture was collected at 48 h post-transfection and then serially passaged on MARC-145 cells. The cytopathic effect (CPE) was observed daily. Rescued viruses were confirmed by an IFA using PRRSV N protein-specific monoclonal antibodies (prepared and stored in our laboratory).

### *In vitro* growth kinetics of the rescued viruses

To analyze the *in vitro* growth characteristics, monolayers MARC-145 or primary PAMs were individually infected with the rescued chimeric virus at a multiplicity of infection (MOI) of 0.1. Virus titers in cell cultures were determined by a microtitration infectivity assay and recorded as 50% tissue culture infective dose per milliliter (TCID_50_/mL) using the Reed-Muench method in MARC-145 cells, with a detection limit of 10^2^ TCID_50_/mL. Briefly, cells were plated in 96-well plates and inoculated with virus suspensions (100 μL/well) prepared by serial 10-fold dilution with DMEM containing 5% FBS. Plates were incubated for 48 hours and the virus was determined by IFA. All assays were repeated three times independently.

### Animal trials for the pathogenicity and protection analysis of the rescued chimeric viruses

To evaluate the pathogenicity and protection of the chimeric virus, thirty-six 29-day-old SPF Landrace pigs which were confirmed to be negative for PRRSV nucleic acid and antibodies, were randomly divided into six groups as shown in **Figure 2**. Each group was separately raised in isolated room. The pig in each group was intramuscularly inoculated with 2 mL of chimeric virus diluted to 10^5^ TCID_50_/mL or 2 mL DMEM medium as a negative control. At 28 dpv, pigs in each group were intranasally inoculated with 2 mL of CHsx1401 virus diluted to 10^5^ TCID_50_/mL or 2 mL DMEM medium as the negative control. Clinical signs, including rectal temperature and respiratory disease, were recorded every other day and scored as previously described (49). Pigs were weighed weekly to calculate the average daily gain (ADG). Serum samples were also collected at 0, 3, 5, 7, 10, 14, 21, 28 dpv and 0, 3, 5, 7, 10, 14, 21 dpi for the titration of viremia by a microtitration infectivity assay and detection of antibodies specific for PRRSV N protein by using commercial enzyme-linked immunosorbent assay (ELISA) kits (JNT, #JX60415). The level of antibody is expressed as the sample/positive value (S/P) ratio. A ratio of ≥0.4 was considered seroconversion. Two pigs per group, excluding the positive control, were randomly selected, euthanized, and necropsied at 28 dpv, and all surviving pigs were euthanized and necropsied at 21 dpi. The euthanasia process was conducted following the provisions outlined in the State Standard of the People’s Republic of China (GB/T 42304-2023). In detail, pigs were electrically stunned with 1.3A electric current for 3s, followed by axillary artery bloodletting to death. Necropsy and gross pathological evaluation were performed immediately after euthanize and scored as previously described (49, 50). Specifically, gross lesions were scored from 0 to 100 according to the lesion size of each lung lobe. Each accessory lobe scored 5 points, while the right and left caudal lobes scored 27.5 points each (15 for dorsal and 12.5 for ventral). Lung tissues were excised and fixed in 4% paraformaldehyde for microscopic pathology as well as immunohistochemistry (IHC), and blindly scored from 0 to 4 according to the severity of interstitial pneumonia (0, no microscopic lesions; 1, mild; 2, moderate multifocal interstitial pneumonia; 3, moderate diffusive interstitial pneumonia; 4, severe interstitial pneumonia) or the number of positive cells (0,  no positive cells; 1, 1–10 positive cells; 2, 11–30 positive cells; 3, 31–100 positive cells; 4, ≥100 positive cells) (50). Meanwhile, the viral RNA loads of lung, tonsil, ILNs, and SLNs were quantified by qPCR using primers targeting the M protein coding region. Tissues were crushed and total RNA was extracted from tissues using MagZol Reagent (Magen, #R4801) according to the manufacturer’s instructions, and then 2μg RNA was reverse transcribed into cDNA using FastKing RT kit (TIANGEN, #KR116). A standard curve was generated by plotting the log_10_ copy number of the M gene against the cycling threshold (CT) value, and qPCR was performed using the FastKing One Step RT-qPCR Kit (TIANGEN, # FP314) according to the manufacturer’s instructions. Primers used for RT-qPCR are listed in **Table 1**.

### Statistical analysis

Data were expressed as means ± standard deviations. The significance of the variability was determined by two-way ANOVA multiple comparisons using GraphPad Prism (version 9.0) software. Asterisks indicate statistical significance; NS, no significance; *, *P* < 0.05; **, *P* < 0.01; ***, *P* < 0.001; ****, *P* < 0.0001.

## Data Availability

The datasets presented in this study can be found in online repositories. The names of the repository/repositories and accession number(s) can be found in the article/supplementary material.
